# Psychosocial Assessment Using Telehealth in Adolescents and Young Adults With Cancer: A Partially Randomized Patient Preference Pilot Study

**DOI:** 10.2196/resprot.8886

**Published:** 2018-08-29

**Authors:** John Alexander Chalmers, Ursula Margaret Sansom-Daly, Pandora Patterson, Geoffrey McCowage, Antoinette Anazodo

**Affiliations:** ^1^ Cancer Centre for Children Children’s Hospital at Westmead Westmead Australia; ^2^ Discipline of Paediatrics School of Women’s and Children’s Health, UNSW Medicine University of New South Wales Sydney Australia; ^3^ Behavioural Sciences Unit Kids Cancer Centre Sydney Children’s Hospital Randwick Australia; ^4^ Sydney Youth Cancer Service Prince of Wales Hospitals Randwick Australia; ^5^ Department of Research, Evaluation & Social Policy CanTeen Australia Newtown Australia; ^6^ Cancer Nursing Research Unit University of Sydney Sydney Australia; ^7^ Nelune Comprehensive Cancer Centre Prince of Wales Hospitals Randwick Australia; ^8^ Kids Cancer Centre Sydney Children’s Hospital Sydney Australia

**Keywords:** telehealth, videoconferencing, psychosocial, psychological assessment, adolescent and young adult, cancer

## Abstract

**Background:**

Adolescent and young adults with cancer are at increased risk of psychosocial difficulties relative to their healthy peers. Current models of inpatient face-to-face psychosocial care might limit the capacity for clinicians to provide timely and personalized assessment and intervention for this group. Telehealth offers a promising alternative toward increasing access to the provision of evidence-based psychosocial assessment and treatment for adolescent and young adults with cancer.

**Objective:**

This pilot study aimed to assess the feasibility and acceptability for both patients and clinicians of providing a psychosocial assessment via telehealth to adolescents and young adults currently receiving treatment for cancer, relative to face-to-face delivery.

**Methods:**

We included patients who were aged 15-25 years, currently receiving treatment, could speak English well, and medically stable. Patients were recruited from oncology clinics or wards from 5 hospitals located across Sydney and Canberra, Australia, and allocated them to receive psychosocial assessment (Adolescent and Young Adult Oncology Psychosocial Assessment Measure) with a clinical psychologist or social worker through face-to-face or telehealth modalities using a partially randomized patient preference model. Patients completed a pre- and postassessment questionnaire comprising validated and purposely designed feasibility and acceptability indices, including the impact of technical difficulties, if patients had their own devices; number of patients who were content with their group allocation; self-reported preference of modality; Treatment Credibility and Expectations Questionnaire; and Working Alliance Inventory. Clinicians also completed a postassessment questionnaire rating their impressions of the acceptability and feasibility of intervention delivery by each modality.

**Results:**

Of 29 patients approached, 23 consented to participate (response rate: 79%). Participants were partially randomized to either telehealth (8/23, 35%; mean age 16.50 years, range 15-23 years; females: 4/8, 50%) or face-to-face (11/23, 62%; mean age 17 years, range 15-22 years; females: 8/11, 72%) conditions. Four participants withdrew consent because of logistical or medical complications (attrition rate: 17.4%). Most participants (6/8, 75%) in the telehealth group used their computer or iPad (2 were provided with an iPad), with minor technical difficulties occurring in 3 of 8 (37.5%) assessments. Participants in both groups rated high working alliance (Working Alliance Inventory; median patient response in the telehealth group, 74, range 59-84 and face-to-face group, 63, range 51-84) and reported positive beliefs regarding the credibility and expectations of their treatment group. Postassessment preferences between face-to-face or telehealth modalities varied. Most patients in the telehealth group (5/8, 63%) reported no preference, whereas 6 of 11 (55%) in the face-to-face group reported a preference for the face-to-face modality.

**Conclusions:**

Telehealth is acceptable as patient comfort was comparable across modalities, with no significant technological barriers experienced. However, patients varied in their preferred interview modality, highlighting the need to tailor the treatment to patient preference and circumstances.

**Trial Registration:**

Australian New Zealand Clinical Trials Registry ACTRN12614001142628; https://www.anzctr.org.au/Trial/Registration/TrialReview.aspx?id=366609 (Archived by WebCite at http://www.webcitation.org/721889HpE)

## Introduction

There has been growing recognition that psychosocial needs of adolescent and young adult (AYA) patients with cancer aged 15-25 years are different from both adults and younger children and warrant specialized services, including timely psychosocial assessment [1,2]. A cancer diagnosis during adolescence has the potential to significantly impact many aspects of normal development, including physical, psychological, social, sexual, educational, and financial domains [3]. In addition, the AYA years are a time during which individuals are at an increased risk of developing mental health disorders, even without the severe stress of a cancer diagnosis [4]. The combination of these factors means that compared with other age groups, young people with cancer often experience more complex psychological distress and social challenges, which might require more long-term, time-intensive psychosocial assessment and intervention [5-10]. Recent studies have reported that clinical-level distress is observed in 23%-27% of AYAs within the first year postdiagnosis [6,8]. Without appropriate assessment and intervention, this might translate into serious long-term mental health risks. A recent study reported that AYAs with cancer are at a significantly higher risk of suicidal behavior (both attempts and completed attempts) in the year after, and up to 5 years postdiagnosis, compared with their peers without cancer [10].

Besides being associated with a poorer quality of life in its own right [11], elevated distress among AYAs with cancer is also likely to have adverse impact on other clinical factors, such as treatment adherence [12], and their ability to communicate effectively about their symptoms, which might delay or exacerbate treatment complications [13,14]. In addition, distress contributes to delays in the postcancer treatment of AYAs reintegrating into normal life, potentially affecting schooling or work and relationships for years into survivorship [15,16]. Ensuring that AYAs with cancer suffering from elevated distress have access to the services they need (eg, psycho-oncology) at the time they need it is therefore important.

AYAs with cancer have unique psychosocial needs [17], and the provision of quality care needs to respect their preferences and provide appropriate emotional support, information, and physical comfort. The experience AYAs have with their care affects their functional, emotional, and social adjustment [18]. The provision of timely psychosocial assessment and intervention for AYAs with cancer is another fundamental component of best practice care [3,19] and is recommended as part of gold standard AYA clinical care in several international jurisdictions, including the UK and Australia [20]. However, considerable barriers exist for this group of patients accessing assessment and intervention. Although Australian rural and remote patients with cancer often need to travel long distances for specialist care, at great expense, time, and inconvenience to their jobs and family [21-23], AYAs are at a particular disadvantage. Many hospitals lack specialist AYA services or health professionals with AYA expertise, and the available specialized services are typically located in metropolitan centers [24,25]. AYAs are a dispersed population, and sometimes live a great distance from their treating hospital. These barriers sometimes mean patients avoid traveling for less “urgent” or compulsory aspects of their care altogether (such as receiving psychosocial assessment or intervention), potentially affecting clinical outcomes.

One method of ensuring higher access to appropriate psychosocial assessment and intervention in AYA patients with cancer is telehealth. Telehealth “involves the use of modern information technology, especially two-way interactive audio/video communications, computers, and telemetry (ability to exchange data), to deliver health (and mental health) services to remote patients” [26]. Previous reviews have highlighted that telehealth might allow for the provision of specialist assessment and intervention to previously inaccessible and remote populations. Telehealth is also valuable in urban areas, especially for those who find it difficult to travel because of logistical or treatment-related constraints. Moreover, providing care using telehealth stands to benefit health providers, as Web-based consultations might reduce clinicians’ travel time costs when caring for patients in diverse locations and increase the efficiency of limited clinician resources, facilitating an increased capacity in service delivery [27]. Finally, AYAs can be difficult to engage in psychosocial services because of barriers such as the stigma of accessing mental health services [28]. Telehealth offers flexibility to the delivery modality of psychosocial care and might assist in enhancing their engagement, by allowing them to talk to a mental health clinician in a truly private setting, and not being seen by a clinician in a ward or an outpatient clinic.

A key part of providing quality psychosocial care to AYAs is regular psychosocial screening and assessment, which enables members of the health care team to identify patients in distress, as well as those at risk of poor psychosocial outcomes, while detecting specific unmet needs that could be exacerbating patient distress. Ongoing assessment throughout treatment ensures unmet needs are addressed, even when those needs change [2,29]. Telehealth offers a promising avenue to ensure that adequate psychosocial assessment is delivered to AYA patients at appropriate time points throughout their cancer journey and into survivorship.

At present, no research has specifically examined the feasibility and acceptability of using telehealth to provide psychosocial assessment for AYAs with cancer. However, in the intervention literature, several studies have suggested that AYA patients might be amenable to receiving psychosocial assessment through telehealth [30,31]. The provision of psychosocial support using telehealth in adult patients with cancer has been found to be satisfactory and feasible [32]. Telehealth interventions can achieve therapeutic alliance and rapport (ie, the relational bond between a clinician and a patient) equivalent to face-to-face therapy [33], and telehealth interventions for childhood and AYA cancer survivors implemented by phone, website, and Facebook have demonstrated feasibility and acceptability [34-38]. Finally, telehealth interventions have shown similar efficacy to face-to-face intervention in improving the quality of life of cancer survivors [39]. Despite these promising findings, no previous studies have investigated the feasibility or acceptability of using telehealth technology to provide psychosocial assessments to AYA patients regarding the treatment of cancer; this is critical to establish given that the literature to date has been mixed regarding whether telehealth is an appropriate, acceptable, or feasible modality through which psychologically distressed patients’ needs can (or should) be met [40,41]. As part of one of the first clinical consultations during which a patient’s distress, illness adjustment, and mental health history might be fully explored, establishing the feasibility and acceptability of telehealth for psychological assessment is crucial to future development and expansion of AYAs’ access to these services as part of best practice clinical care.

This study aimed to evaluate the feasibility and acceptability of telehealth-delivered psychosocial assessment among AYAs undergoing treatment for cancer compared with patients receiving face-to-face assessment. Both patients and clinicians provided feedback for this evaluation. Specifically, the objectives were to assess the feasibility of using telehealth to deliver psychosocial assessments to AYAs during cancer treatment by examining the frequency of technical difficulties experienced in the telehealth group and how many patients in the telehealth group had access to their own devices compared with how many required one. The acceptability of using telehealth to deliver psychosocial assessments to AYAs during cancer treatment was determined by examining (1) how many patients were content with their group allocation and how many patients requested the alternative modality; (2) patients’ subjective frustration with technical difficulties if or when these occurred in the telehealth condition; (3) self-reported preference of modality for all patients and to explore reasons for the stated preference; (4) patient outcome expectations and credibility beliefs about receiving the psychosocial assessment via telehealth or face-to-face; (5) patient-reported levels of working alliance across both telehealth and face-to-face; and (6) clinicians’ impressions of patients’ engagement, comfort, rapport, and openness across both modalities. We hoped that indexing these feasibility and acceptability indices could directly inform the design and planning of a future, larger randomized trial of telehealth-delivered psychosocial assessment for AYA patients with cancer across Australia.

## Methods

### Participants

This study was approved by the Sydney Children’s Hospitals Network Human Research Ethics Committee. We recruited patients (both inpatients and outpatients) from the following 5 sites in the state of New South Wales (NSW), Australia: Westmead Hospital and The Children’s Hospital at Westmead (Western Sydney), Prince of Wales Hospital and Sydney Children’s Hospital (East Sydney), and Canberra Hospital (Canberra). While the 5 sites are situated in urban centers, they capture both urban and rural patients. Patients were eligible to participate in this study if they had a cancer diagnosis, were on treatment, and were aged 15-25 years. Patients presenting with skin cancer diagnoses were ineligible to participate. Consistent with the New South Wales Health Policy and the clinical care model provided within the AYA services the study recruited from, patients aged <18 years were invited to provide their consent, independent of a legal parent or guardian. The additional inclusion criteria included speaking and being able to read English well and being medically stable. Patients were determined to be medically stable using clinical judgment, liaising with the treating team as appropriate. If patients in the telehealth group did not have access to a necessary device (eg, a computer or an iPad), they were provided one. No compensation was offered for participation in this study. Furthermore, 3 clinicians were involved in delivering the study, one at each site—two clinical psychologists and one social worker.

### Design

This pilot study was a partially randomized patient preference trial (ACTRN12614001142628) [42-44], which allowed participants to opt out of the randomized allocation and choose their preferred group. This design enables individual differences or biases to be accounted for among those who elect to be randomized, while also being better in assessing the manner in which clinical services are offered and selected by patients in real clinical contexts. We randomized patients who consented to participate in the study to receive their psychosocial assessment via face-to-face or telehealth modalities. If patients were not comfortable being randomized, they were offered the choice to select their preferred assessment modality.

### Procedure

A Masters-level clinical psychologist or social worker approached patients in person bedside or before or after a clinic appointment for introducing the study and providing the study information form. This necessitated patients having some degree of face-to-face contact with the clinician before the assessment, regardless of the group allocation. The clinicians had limited previous experiences with using telehealth in a clinical setting and were provided training in the use of telehealth and the software before using it with patients. Following informed consent, patients indicated whether they were comfortable being randomized to either condition. Based on their decision, patients were either randomized (using a simple randomization method with random numbers generated by independent personnel) or chose to receive their psychosocial assessment via face-to-face or telehealth. Patients then completed a preassessment questionnaire battery.

Next, a Masters-level clinical psychologist or social worker assessed patients within 4 weeks using the AYA Oncology Psychosocial Assessment Measure, developed by CanTeen [45]. Patients allocated to telehealth were joined to Web-based videoconferencing software (Cisco Webex), a secure, password-protected videoconferencing application. Immediately following the assessment, care plans were developed collaboratively with patients, as per the AYA Oncology Psychosocial Care Manual [45].

Then, patients immediately completed a postassessment questionnaire battery. Both pre- and postassessment batteries were completed either on paper or online, depending on the patient’s preference. If a patient was identified to be experiencing elevated distress or was assessed to be at acute risk (eg, experiencing suicidal ideation with intent or plan), the clinician responded as appropriate depending on the level of risk and the modality through which the patient was being assessed. This study utilized the same risk management protocol as reported in other recent Australian trials using videoconferencing, which have been shown to be appropriate in terms of screening for and managing mental health risks [41]. Specifically, if assessed face-to-face, risk management procedures were in keeping with standard clinical care (eg, walking patient to emergency, contacting a general practitioner [GP] or community crisis team etc). In contrast, risk management strategies for patients assessed via telehealth were determined by the level of risk. The protocol included actions such as confirming the AYAs’ location and contacting 000 (Australian emergency phone number), liaising with the AYA’s GP or community crisis team, contacting the AYA’s primary caretaker and treating team, and providing the AYA with crisis telephone numbers to contact if they begin to feel less safe and encourage them to talk to their GP.

### Psychosocial Assessment

The AYA Oncology Psychosocial Assessment Measure [45] is a modified version of the HEADSS (Home, Education/Employment, peer group Activities, Drugs, Sexuality, and Suicide/depression) assessment [46], a widely used adolescent psychosocial assessment measure administered by semistructured clinical interview, adapted to suit the circumstance and needs of AYA patients with cancer. It includes the assessment of home environment, education or employment status, social history, drug or alcohol use, sexuality, and mental health status (eg, suicidal or depressed) [45]. Furthermore, the Psychosocial Measure informs the development of a care plan with patients, which might involve ongoing psychosocial support or referral to appropriate services [45].

### Measures

#### Demographics

The preassessment questionnaire battery included demographic information, including age, gender, employment status, diagnosis, and treatments received. We included the Youth Satisfaction Questionnaire [47], measuring satisfaction with overall psychosocial care, in the preassessment battery; this measure has adequate internal consistency [47] and has previously been used among AYAs with cancer [48]. Scores range from 3 to 9, with higher scores denoting higher patient satisfaction.

#### Feasibility

Patients were asked to report whether technical difficulties occurred, and if so, how many minutes of interruption these caused. Our benchmark for feasibility was the resolution of any technical difficulties within 5 minutes (assessed in the postassessment battery).

Patients in the telehealth group also reported whether they had their own device or not. Our benchmark for feasibility was for ≥75% patients to have their own device (assessed in the postassessment battery).

In terms of recruitment rate and attrition, our benchmark for feasibility was for >50% patients approached to consent and participate in this study (assessed in the postassessment battery).

#### Acceptability

We used the number of patients who were content with their group allocation versus the number of patients who requested an alternative modality as an index of acceptability. Our benchmark for acceptability was for 80% patients to be content with their group allocation (assessed in the postassessment battery).

Patients were asked to rate how frustrated they were if they experienced technical difficulties on a scale of 1-10, where an average rating of ≤3 was deemed acceptable (assessed in the postassessment battery).

All patients indicated self-reported preference of modality, or whether they had no preference either way, and were then asked to explain their preference in an open-ended written response. Our benchmark for acceptability was for >80% patients in the telehealth group to prefer their allocation over face-to-face or otherwise have no preference (assessed in the postassessment battery).

Patient outcome expectations and credibility beliefs about receiving the psychosocial assessment via telehealth or face-to-face were assessed with the Treatment Credibility and Expectations Questionnaire [49] in the preassessment battery. In addition, items were modified for this study as appropriate; for example, “treatment” was replaced with “consultation over the internet.” This measure has high internal consistency and includes 6 items, of which, 4 employ a 9-point Likert scale ranging from 1 to 9 and 2 employ an 11-point Likert scale ranging from 0% to 100%; higher scores reflect higher credibility or expectations. Our benchmark for acceptability was for outcomes on this measure to be comparable across groups (assessed in the preassessment battery).

Patients reported levels of working alliance across both telehealth and face-to-face assessed using the Working Alliance Inventory (WAI) [50], which measured AYAs’ perceptions of their relationship (ie, rapport and feeling understood) with the clinician who conducted the assessment. This measure employs a 7-point Likert scale ranging from 1 (not at all) to 7 (exactly), where the total score ranges from 12 to 84, with higher scores reflecting a stronger working alliance. Our benchmark for acceptability was for outcomes on this measure to be comparable across groups (assessed in the postassessment battery).

Upon completion of this study, we asked patients to rate how beneficial and how burdensome the study was respectively, on a scale ranging from 1 (not at all) to 5 (very much; assessed in the preassessment battery).

Clinicians’ impressions of patients’ engagement, comfort, rapport, and openness were assessed across both modalities, whereby clinicians completed a postassessment questionnaire asking them to rate their impression of patients’ engagement, comfort, rapport, and openness on a 10-point Likert scale ranging from 1 (low) to 10 (high). We defined acceptability as outcomes on this measure being commensurate across groups. Our definitions of feasibility and acceptability are similar to other work assessing feasibility and acceptability of telehealth-delivered psychosocial care [51] (assessed in the postassessment battery).

#### Psychosocial State

The preassessment battery included measures of current psychosocial functioning used to characterize the sample, including the Kessler Psychological Distress Scale 10 [52], measuring anxiety and depression symptoms in the past 4 weeks. Scores in this measure range from 10 to 50, where scores <20 indicate normal functioning, 20-24 indicate mild difficulties, 25-29 indicate moderate difficulties, and >30 indicate severe difficulties [52,53]. In addition, patients completed the Pediatric Quality of Life Inventory for adolescents and young adults [54], measuring AYA cancer-specific quality of life. Scores range from 0 to 100, with higher scores indicating a higher quality of life.

### Statistical Analysis

Data were analyses using SPSS Version 24.0 (IBM Corp, Armonk, NY, USA). We calculated descriptive statistics for all measures included. When the data is nonnormally distributed, medians as ranges are reported as a measure of central tendency. Between-group quantitative analyses were not conducted because of the small sample size and associated limited power.

## Results

### Participants

**Figure 1:**
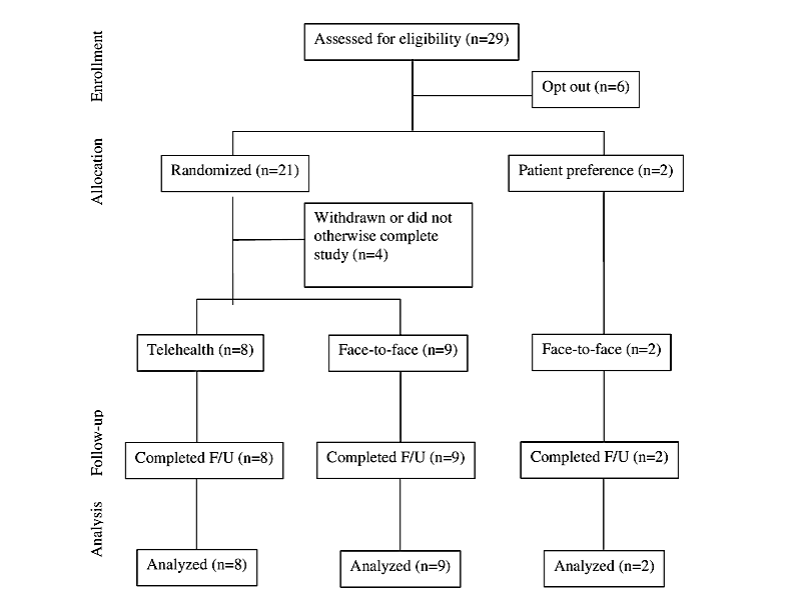
The Consolidated Standards of Reporting Trials (CONSORT) flowchart of participants. F/U: follow-up.

Of the 29 patients approached, 23 (79%) consented to participate and 6 (21%) declined as they were not interested in participating. In addition, 17% (4/23) participants did not complete the study following consent—2 were lost to follow-up, 1 changed mind, and 1 became too unwell and was withdrawn from the study by the investigators (this occurred following consent, but prior to any further participation in the study). Of the 19 remaining participants, 17 were randomized to either telehealth (8/17, 47%) or face-to-face (9/17, 53%) conditions, with 2 participants electing to be assessed face-to-face (Figure 1). The 2 patients who chose the face-to-face modality were collapsed in the data with other face-to-face group participants and not reported separately; this decision was made because of the small number (n=2) of patients who opted out of randomization.

Table 1 summarizes key patient characteristics. In this study, 19 participating AYAs represented a diverse range of ages, gender, employment status, diagnosis, and treatment. All patients were undergoing treatment for their first diagnosis (ie, had not relapsed). AYAs lived a median distance of 24 (range 13-414) km from their treating hospital. As a group, AYAs’ average levels of distress according to Kessler Psychological Distress Scale 10 were in the “normal” range, although some in each group showed elevated distress (telehealth: 2/8, 25%; face-to-face: 3/11, 27%). In addition, our sample reported health-related quality of life scores in the moderate range, although their emotional or physical functioning was somewhat lower. Overall, AYAs reported very high satisfaction with their psychosocial care to date (median Youth Satisfaction Questionnaire score 8.5, range 6-9).

### Feasibility

Technical difficulties occurred during a minority of assessments in the telehealth group (3/8, 37.5%); however, in each case, problems were resolved in <5 minutes, meeting our benchmark of feasibility. The technical difficulties that occurred were all because of inconsistent connection speeds in the context of patients’ devices not being connected to Wi-Fi but relying on the 4G mobile connection. Of telehealth participants, 6 of 8 (75%) used their own computer or iPad and the other 2 (25%) were provided with an iPad as their assessment occurred during an inpatient stay, which met our benchmark of feasibility. Of all 29 patients approached, 19 (65.5%) consented to participate and also completed all components of the study, above our feasibility target of 50%.

### Acceptability

Almost all (17/19, 89%) patients were content with their group allocation, above our acceptability target of 80%. In the telehealth group, all 3 patients who experienced technical difficulties rated their level of frustration because of this as 1 of 10, below our benchmark of 3/10. Table 2 outlines patients’ experiences of the psychosocial assessment. Following the psychosocial assessment, 7 of 8 (87.5%) patients in the telehealth group reported a preference for telehealth or otherwise no preference between face-to-face or telehealth modalities (above our acceptability target of 80%). Evaluating differences in the modality preference across gender, among young men, 3 preferred face-to-face, 3 preferred telehealth, and 1 cited no preference. Among young women, 5 preferred face-to-face, 1 preferred telehealth, and 6 cited no preference. Table 3 outlines qualitative patients’ responses accounting for their reported preference of the assessment modality. Patients reported their perceptions of the ease and comfort associated with each modality, as well as concerns about confidentiality and familiarity with the clinician conducting the interview. Table 4 outlines patients’ expectations of the telehealth or face-to-face modality and their beliefs about the credibility of the modality. Overall, patients had similarly positive expectations for both telehealth and face-to-face modalities and rated high credibility. The median patient response on WAI was 74 (range 59-84) for the telehealth group and 63 (range 51-84) for the face-to-face group. WAI scores for young men (median 67, range 54-81) and young women (median 67, range 51-84) were comparable. In both groups, the median response to whether the study was burdensome was 1 (ie, not at all). The median response to whether the study was beneficial was higher in the telehealth group. Table 5 outlines clinicians’ reports of patients’ engagement, comfort, rapport, and openness. Overall, clinicians tended to report high levels of patient engagement and comfort across both the telehealth and face-to-face groups. Furthermore, clinicians’ ratings of technical difficulties were commensurate with patients’ ratings.

**Table 1:** Patient characteristics.

Variable			Telehealth (n=8)	Face-to-face (n=11)
Age (years), median (range)			16.50 (15-23)	17 (15-22)
Gender (female), n (%)			4 (50)	8 (73)
Live at home (yes), n (%)			8 (100)	11 (100)
Parents separated or divorced (yes), n (%)			2 (25)	3 (27)
Employed, n (%)			4 (50)	6 (54.5)
Speak another language other than English at home (yes), n (%)			2 (25)	3 (27)
**Highest level of education completed, n (%)**				
	Year 10 or below		5 (62.5)	5 (45)
	Technical and Further Education certificate		0 (0)	2 (18)
	Year 12		3 (37.5)	4 (36)
Kilometers living from hospital, median (range)			22.50 (13-414)	24 (17-94)
**Remoteness index^a^**				
	Major cities		7 (87.5)	10 (91)
	Inner regional		1 (12.5)	1 (9)
**Treatment factors**				
	**Diagnosis, n (%)**			
		Acute lymphoblastic leukemia	0 (0)	1 (9)
		Acute myeloid leukemia	0 (0)	2 (18)
		Brain cancer	0 (0)	1 (9)
		Hodgkin’s Lymphoma	1 (12.5)	4 (36)
		Non-Hodgkin’s Lymphoma	1 (12.5)	1 (9)
		Sarcoma of the bone	1 (12.5)	0 (0)
		Soft tissue sarcoma	3 (37.5)	1 (9)
		Other	2 (25)	1 (9)
	Age at diagnosis, median (range)		16 (14-23)	16 (13-21)
	Received chemotherapy, n (%)		6 (75)	9 (82)
	Received radiotherapy, n (%)		1 (12.5)	4 (34)
	Received surgery, n (%)		2 (25)	2 (18)
	Bone-marrow transplant, n (%)		2 (25)	1 (9)
	Youth Satisfaction Questionnaire, median (range)		9 (6-9)	8 (7-9)
**Functioning**				
	Kessler Psychological Distress Scale 10^b^, median (range)		17.50 (13-27)^c^	17 (12-37)^d^
	**Pediatric Quality of Life Inventory for adolescents and young adults^e^, mean (SD)**			
		Total score	66.75 (14.84)	61.86 (14.90)
		Physical health	39.84 (17.82)	42.33 (19.93)
		Emotional	65.00 (22.36)	60.91 (18.14)
		Social	83.75 (11.57)	77.73 (12.72)
		Study or work	53.13 (26.98)	65.45 (26.31)
		Pain and hurt	59.38 (17.36)	54.46 (17.02)
		Nausea	61.88 (28.53)	58.18 (25.03)
		Procedural anxiety	81.25 (29.12)	75.76 (31.94)
		Treatment anxiety	80.21 (16.02)	68.94 (26.64)
		Worry	65.63 (24.98)	46.21 (29.67)
		Cognitive problems	71.88 (21.87)	61.82 (31.09)
		Physical appearance	87.50 (14.77)	64.40 (34.17)
		Communication	82.29 (17.50)	71.97 (31.02)
In general, would you say your health is 1 (excellent)-5 (poor), median (range)			3 (2-4)	3 (2-3)
Before your diagnosis, had you ever seen a psychologist, social worker, counselor or psychiatrist at any time in the past (yes), n (%)			2 (25)	6 (54.5)

^a^The Australian Statistical Geography Standard Remoteness Structure.

^b^Scores range from 10 to 50, where higher scores indicate higher psychological distress.

^c^Two patients fell in the clinically elevated range (ie, moderate to severe, scores above 24).

^d^Three patients fell in the clinically elevated range (ie, moderate to severe, scores above 24).

^e^Scores range from 0 to 100, where higher scores indicate a higher quality of life.

**Table 2:** Patients’ experience of psychosocial assessment (assessed in the postassessment battery).

Question/statement and response			Telehealth (n=8)	Face-to-face (n=11)
**Were there any topics that you did not feel comfortable discussing with the psychologist during this consultation, n (%)**				
	Yes		1 (12.5)	0 (0)
	No		7 (87.5)	11 (100)
**Were you happy having your assessment online or in person or would you have preferred doing it the other way, n (%)**				
	Prefer online		1 (12.5)	3 (27.3)
	Prefer face-to-face		2 (25)	6 (54.5)
	No preference		5 (62.5)	2 (18.2)
Please rate your level of comfort in talking about personal issues online or in person (compared with in person or online)^a^, median (range)			8.50 (3-10)	8.50 (6-10)
The consultation with the psychologist or social worker did not take too long to complete^b^, median (range)			2 (1-4)	2 (1-3)
I did not have to wait too long for my consultation with the psychologist or social worker^b^, median (range)			1.50 (1-2)	2 (1-2)
It was difficult to travel to my consultation with the psychologist or social worker^b^, median (range)			4.50 (1-5)	4 (1-5)
The questions in the psychological consultation were easy to understand^b^, median (range)			1.50 (1-2)	2 (1-3)
The consultation with the psychologist or social worker covered issues that were relevant to me^b^, median (range)			1.50 (1-2)	2 (1-2)
I would have liked to have completed my psychological consultation in a more private location, median (range)			4 (1-5)	4 (2-5)
I would be happy to have a psychological consultation again as part of my future care^b^, median (range)			2.50 (1-3)	2 (1-3)
Completing this psychological assessment has helped me to communicate my emotional needs to my medical care team^b^, median (range)			2 (1-3)	2 (1-3)
Face-to-face only: How long did it take you to get to the hospital for your appointment today (min), median (range)			—	30 (20-120)
**Online only: Did you experience any technical difficulties during today’s online consultation^b^, n (%)**				
	Yes		3 (37.5)	—
	No		5 (62.5)	—
**Online only: Technical difficulties; if yes, how long did it take to resolve^b^, n (%)**				
	<5 min		3 (100)	—
	>5 min		0 (0)	—
Online only: Technical difficulties; if yes, how much did these technical difficulties frustrate you^a^, median (range)			1 (1-1)	—
Was participation in this study burdensome to you in any way^c^, median (range)			1 (1-2)	1 (1-4)
Was participation in this study beneficial to you in any way^c^, median (range)			4 (3-5)	1.5 (1-5)

^a^Response set: 1-10.

^b^Response set: 1 (strongly agree)-5 (strongly disagree).

^c^Response set: 1 (not at all)-5 (very much)

**Table 3:** Qualitative patient reports explaining their preference of modality for either telehealth, face-to-face, or no preference (assessed in the postassessment battery).

Preference		Quote
**Telehealth**		
	Prefer online	“Because it’s a lot easier as I can just have my session done when I’m at home.”
	Prefer face- to-face	“Because you want to familiarize who they are first and how they can help you.” “Face-to-face for me allows for deeper discussion, I felt slightly removed from the whole thing when doing it online. It is also a better setting face-to-face as I was aware of my housemate being around which limited some of the topics that I would talk about.”
	No preference	“I do not have a preference as I personally believe that it didn’t make a difference to our conversation.” “Personally, speaking to somebody via technology achieves the same result as speaking face-to-face.” “I’m comfortable in both situations.” “Either way works for me.”
**Face-to-face**		
	Prefer online	“Yeah, only reason I prefer online is because my social worker is too far from my home.” “Because I live far away.”
	Prefer face-to-face	“Easier.” “I just find it easier.” “Feel more comfortable discussing situations in person.” “It’s good to talk to someone face-to-face.” “I prefer the face-to-face because I feel it more comfortable to talk to an actual person.” “Easier to talk to.”
	No preference	“I don’t mind either way.” “I think both are as good as each other.”

**Table 4:** Treatment credibility and expectations questionnaire [49] (completed preassessment).

Question		Median (range)	
		Telehealth^a^ (n=8)	Face-to-face^a^ (n=11)
At this point, how logical does it seem to you to have a consultation over the internet or in person?		6.50 (2-9)	7 (2-9)
At this point, how successfully do you think this consultation over the internet or in person will be in helping you cope?		6.50 (4-9)	6 (1-9)
How confident would you be in recommending having a consultation with your psychologist over the internet or in person?		7 (4-9)	7 (1-9)
By the end of your consultation over the internet or in person, how much better do you think you will feel about your current situation?		70 (40-80)	70 (0-90)
At this point, how much do you really feel that your consultation over the internet or in person will help you cope with your current situation?		6.50 (3-9)	6 (1-9)
By the end of the consultation over the internet or in person, how much improvement in how you are coping with your situation do you really feel will occur?		55 (40-90)	60 (0-90)

^a^Four items employ a 9-point Likert scale ranging from 1 to 9, and 2 items employ an 11-point Likert scale ranging from 0% to 100%; higher scores reflect higher credibility or expectations.

**Table 5:** Clinicians’ ratings (n=3) of clinicians’ experience of psychosocial assessment (completed postassessment).

Question	Telehealth (n=8)	Face-to-face (n=11)
Please rate how active or vocal the patient was during the assessment today, median (range)	9 (6-10)	8 (4-10)
How comfortable did you feel conducting the assessment today, median (range)	9 (7-10)	9 (6-10)
Rate how good the rapport was between yourself and the AYA^a^ patient overall, median (range)	9 (7-10)	8 (6-9)
How open did you feel the AYA patient was, median (range)	8 (6-10)	7 (4-10)
Online only: Did any technical difficulties arise during this session (yes), n (%)	3 (37.5)	—
If yes, how long did it take less than 5 minutes to resolve (yes), n (%)	3 (100)	—

^a^AYA: adolescent and young adult.

## Discussion

### Principal Findings

This study explored whether the administration of a psychosocial assessment would be both feasible and acceptable to AYAs currently receiving cancer treatment if delivered using telehealth. Overall, the delivery of psychosocial assessments via telehealth was found to be feasible. We were able to deliver telehealth-based psychosocial assessments to a broad patient group with a range of cancer diagnoses, ages, and varied distress levels and across both young men and women. All patients randomized to the telehealth group had their own device, which they used to connect to their Web-based assessment (unless they were inpatients, in which case a device was provided). Furthermore, while some patients did experience technical difficulties during their assessment, these were resolved quickly and resulted in minimal frustration, which is in keeping with other recent telehealth studies where technical difficulties and associated dissatisfaction were minimal [51,55]. Clinicians’ scores indicated a high degree of acceptability from their perspective as well. It is important to note that clinicians did not have prior experience in using telehealth as a modality in providing clinical care, highlighting that when provided with appropriate training, telehealth is a feasible tool to implement even in settings where clinicians might not have had previous experience in doing so.

Psychosocial assessments delivered via telehealth were also found to be acceptable. The ability to effectively deliver an inherently interpersonal service through the medium of technology has been posed as one of the key ethical tensions involved in the expansion of telehealth services [6,56]. Our findings highlighted that both clinicians and patients felt comfortable with the telehealth modality and reported comparably high levels of patients’ engagement and comfort across telehealth and face-to-face modalities. Critically, patients’ perceptions of the therapeutic collaborative relationship with the psychologist or social worker were also comparable across groups. The high proportion of patients who were willing to be randomized to either condition (telehealth or face-to-face) at the outset further emphasizes that future trials will be able to employ this design with confidence. In the context of the partially randomized patient preference design used in this study, AYAs’ openness to both models of care appears to reflect that both models of undertaking psychosocial assessments for AYA patients with cancer might be developmentally and clinically appropriate in different circumstances. Concerning gender, our results suggested that both males and females might be open to telehealth as a model of care; although our sample was quite small, this aligns with previous findings that report no impact of gender on the acceptability of telehealth technology [57,58].

The qualitative responses showed that patients who preferred telehealth indicated this was largely because of logistical issues, whereby telehealth reduced the burden of travel and increased ease of attending an assessment. Patients who preferred face-to-face assessment indicated it was simply more comfortable and easier to engage with a clinician in person. Although telehealth was the explicit preference among a minority of AYAs, of note, almost three times as many AYAs in the telehealth group expressed having “no preference” of modality. Despite ethical concerns in the literature regarding the potential for confidentiality and security issues in telehealth [40], we found little evidence of AYAs being overly concerned around these confidentiality aspects, and it did not appear to affect our participation rates. Like previous Australian studies, we used a more secure videoconferencing platform, with password-protected sessions, and liaised with patients to ensure that the session was undertaken in a private, confidential location [41,48]. Rather, our findings indicate that while AYAs may have a general tendency to prefer face-to-face interactions with psychosocial clinicians, they may become more open to the possibilities or potential benefits of telehealth once they have tried it for themselves. This notion agrees with previous literature that has found young people exposed to telehealth reported appreciation for the privacy it allows [59], which overcomes barriers to service utilization, such as mental health stigma, and the sense of empowerment and control patients have about terminating sessions [60]. Thus, future studies should investigate whether and how patients’ (and clinicians’) attitude toward telehealth may change with exposure to it. In order to better gauge AYAs’ relative preferences for telehealth versus face-to-face delivered psychosocial services, a future trial employing a crossover design in which AYAs gain exposure to both models of care would be recommended. The variance in patients’ preferences reported in this study (and the high proportion indicating no preference) highlights the importance of clinicians engaging collaboratively with AYAs to ensure that they offer a service that suits each patient’s needs.

Although participants’ preference for assessment modality varied, the median level of reported comfort talking about personal issues was comparable across groups. In addition, participants’ perceptions of the working relationship with their psychologist appeared equally positive regardless of their gender or how far away they lived from the hospital. These findings echo other recent studies that have also found that young people report positive perceptions of psychological interactions that take place using telehealth [61,62]. For many patients, therefore, concerns about the quality of the rapport or working relationship over telehealth might not be the primary driving factor underlying preferences between the two treatment models.

Our sample also represented a group of AYAs with lower total health-related quality of life, and in particular lower emotional and physical functioning, relative to previously reported samples of AYA cancer survivors [11]. Telehealth technologies might be particularly helpful for individuals currently experiencing a great symptom burden and for whom additional travel to a hospital site to receive support would be difficult [18,48]. Indeed, satisfaction with telehealth is understandably very high in rural communities where the need to travel for face-to-face care is prohibitive [63]. As AYA psychosocial assessments are increasingly recognized as a key part of the best practice clinical care (Clinical Oncology Society of Australia guidelines [56]), this study shows the potential utility of incorporating telehealth into that delivery model and suggests that this would be feasible and acceptable to both patients and clinicians.

### Strengths

This study has several strengths. To the best of our knowledge, this is the first study to explore the clinically important issue of whether using telehealth to provide psychosocial assessment is viable among AYAs with cancer. We had a high response rate and level of participation among patients approached to participate, highlighting the appeal and importance of the study to AYAs. Contrary to prior studies where AYAs’ ownership of technology was a requirement for participation [51], we were able to provide AYAs with access to technological equipment if they needed it, ensuring equity of access to AYAs with varied socioeconomic and financial resources. Furthermore, the study demonstrated the feasibility and acceptability of using a telehealth modality to conduct psychosocial assessments among patients of varying levels of psychological distress. Prior work has questioned the appropriateness of using emerging technologies, including telehealth, with distressed populations, citing safety concerns [18,64]. This study provides a first step to supporting the acceptability and feasibility of telehealth psychosocial assessment for vulnerable and distressed groups and is in line with previous work demonstrating that distress can be assessed in clinically and ethically appropriate ways using Web-based telehealth technologies 41.

### Limitations

This study also has several limitations. First, the small sample size and nonnormal data distribution restricted quantitative between-group analyses. Second, all patients received some degree of face-to-face engagement by the clinicians regardless of group randomization, which may have lessened the differences between the two groups. This occurred in the patient recruitment and consent process and was conducted by the assessing clinician because of resource limitations. However, recent research has reported that emerging technologies, such as telehealth, are likely to be best used as “adjuncts” or supplements to routine, face-to-face clinical care [13,14,18,65-68]. As such, the mix of face-to-face and telehealth care our sample received is likely to be a good approximation of how this might be incorporated into routine practice in clinical settings. In future, studies using a “crossover” design would assist in more rigorously comparing the relative feasibility and acceptability of these two modalities, whereby participants would have exposure to both face-to-face and telehealth modalities before providing feedback on these. In addition, although the psychosocial assessment used is manualized and clinicians engaged in self-reported fidelity checks where they ticked off each component of the assessment for each patient, we did not include a rigorous assessment of clinicians’ fidelity to the AYA psychosocial assessment as one of our outcome measures; consequently, it is possible that the face-to-face and telehealth arms also differed in the delivery of this assessment between clinicians. Finally, as a pilot study, this study has inherent limitations with respect to the generalizability of the results without a future larger scale trial being undertaken.

### Conclusions

Despite these limitations, this study represents the first attempt to investigate the acceptability and feasibility of telehealth in providing a psychosocial assessment to AYAs on cancer treatment, as well as clinicians’ experience of patients’ response to telehealth. Overall, telehealth was well received, patient comfort was comparable across modalities, and no significant technological barriers were experienced. While some patients will indicate a preference for a face-to-face assessment, a preference that needs to be respected, telehealth offers a feasible and acceptable alternative for patients who prefer it or otherwise would be burdened by accessing face-to-face assessment.

## Appendix

Multimedia Appendix 1 CONSORT‐EHEALTH checklist (V 1.6.1).

## Abbreviations

AYA: adolescent and young adult
WAI: Working Alliance Inventory
